# Enhancing Near‐Infrared Two‐Photon Absorption of Aza‐Boron‐Dipyrromethene Compounds Through Intramolecular Charge Transfer Via Electron Donating Substitution

**DOI:** 10.1002/cplu.202500354

**Published:** 2025-08-08

**Authors:** Anıl Doğan, Halil Yılmaz, Ahmet Karatay, Elif Akhüseyin Yıldız, Gökhan Sevinç, Huseyin Unver, Bahadir Boyacioglu, Mustafa Hayvali, Ayhan Elmali

**Affiliations:** ^1^ Department of Engineering Physics Faculty of Engineering Ankara University Ankara 06100 Turkiye; ^2^ Department of Chemistry Faculty of Science Ankara University Ankara 06100 Turkiye; ^3^ Institute of Artificial Intelligence Ankara University Ankara TR‐06100 Türkiye; ^4^ Department of Chemistry Science and Literature Faculty Bilecik Şeyh Edebali University Bilecik 11230 Turkiye; ^5^ Department of Physics Faculty of Science Ankara University Ankara TR‐06100 Turkiye; ^6^ Vocational School of Health Services Ankara University Kecioren‐Ankara TR‐06290 Turkiye

**Keywords:** aza‐BODIPY, DFT, intramolecular charge transfer, open aperture z‐scan, two‐photon absorption, ultrafast pump‐probe spectroscopy

## Abstract

The nonlinear optical properties of aza‐borondipyrromethene (aza‐BODIPY) derivatives modified with 4‐methoxyphenyl, 2,4‐dimethoxyphenyl, and 4‐N,N‐diphenylaminophenyl groups at the 1 and 7 positions of the core structure are evaluated. The effects of substituents and solvent polarity (tetrahydrofuran (THF) and chloroform (CHCl_3)_) on the linear absorption and fluorescence properties are systematically investigated. The target aza‐BODIPYs exhibit higher absorption in CHCl_3_ compared to THF. While solvent polarity exerted a minimal effect on the absorption and fluorescence intensity, the fluorescence in **BOD2** and **BOD3** is markedly quenched as a result of the electron‐donating methoxy groups and the pronounced intramolecular charge transfer (ICT) characteristics of the diphenylamine unit. Moreover, all compounds shows strong near‐infrared two‐photon absorption (TPA) behavior. The TPA cross‐sections of **BOD1** and **BOD3** are measured as 61 and 269 GM at 1000 nm, respectively, and **BOD3** showed the highest value due to the enhanced ICT efficiency. Density functional theory calculations are also performed to study the thermodynamic and photophysical properties in different media. The findings indicated an enhancement in molecular stability and a reduction in HOMO–LUMO gaps, particularly in THF. Among the compounds, **BOD3** stood out with its low energy gap and high polarizability, and its theoretical NLO parameters are in strong agreement with the experimental TPA findings.

## Graphical abstract

Aza‐BODIPY compounds is designed with 4‐methoxyphenyl, 2,4‐dimethoxyphenyl, and 4‐N,N‐diphenylaminophenyl groups at the 1 and 7 positions. The effects of substituents and solvent polarity (tetrahydrofuran and chloroform) on the linear absorption, fluorescence behaviors as well as nonlinear absorption properties are investigated. Nonlinear absorption properties and charge transfer dynamics are analyzed by open aperture Z‐scan experiments and ultrafast pump‐probe spectroscopy measurements. The thermodynamic and photophysical properties in both solvents are also determined by Density functional theory calculations.

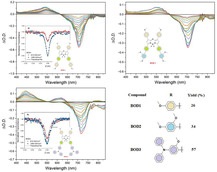

## 1 Introduction

Boron‐dipyrromethene (BODIPY) and its derivatives are widely used in many areas ranging from biomedical imaging to photothermal therapy and optoelectronic systems due to their strong light absorption capacity, high fluorescence quantum yield, photostability, and tunable HOMO–LUMO energy gap. The photophysical properties of these compounds are highly sensitive to small changes in their molecular structures. Specifically, the position of the substituents, most commonly phenyl, and the electron‐donating/withdrawing character of the substituents have a very significant impact on the wavelength of excitation and emission of BODIPY derivatives. Moreover, their high molar absorption coefficient, nonlinear optical (NLO) characteristics, and chemical stability have allowed these compounds to be attractive to a variety of applications,^[^ ^1^, ^2^, ^3^, ^4^ ^–^ ^5^ ^]^ with BODIPY in particular being well known for its high photochemical stability and widespread use in biological monitoring and fluorescence imaging applications.^[^ ^6^, ^7^, ^8^, ^9^ ^–^ ^10^ ^]^ In addition, its flexibility in terms of solubility allows it to be used in different solvent environments.^[^ ^11^ ^,^ ^12^ ^]^ Therefore, BODIPY compounds are of great interest in various applications such as catalysis, imaging, sensors, cancer treatment, and solar cells, due to chemical and photophysical advantages.^[^ ^13^, ^14^, ^15^, ^16^ ^–^ ^17^ ^]^ In 2002, O’Shea et al. first synthesized aza‐BODIPY that is basically BODIPY with a nitrogen atom added in place of a carbon atom in meso (8)‐position of the dipyrromethene core.^[^ ^18^ ^]^ This nitrogen atom changes the electronic structures of the molecule, leading to a shift in the absorption spectrum, usually into the red and near‐infrared (NIR) regions.^[^ ^19^ ^,^ ^20^ ^]^ This shift makes aza‐BODIPY compounds suitable candidates for NIR applications.^[^ ^21^, ^22^, ^23^ ^–^ ^24^ ^]^

Photophysical properties of BODIPY derivatives can be changed by substituting atoms and/or molecule groups. Modifying the electron‐donor/acceptor groups symmetrically (D‐A‐D) or asymmetrically (D‐A), expanding the length of the *π*‐conjugated structure and changing the position and number of substituents are significant methods to modify BODIPY compounds.^[^ ^25^, ^26^, ^27^ ^–^ ^28^ ^]^ These arrangements can provide the higher two‐photon absorption cross‐section (TPCS) compared to the simple BODIPY compounds.^[^ ^29^, ^30^ ^–^ ^31^ ^]^ In addition, it is known that in organic materials where intramolecular charge transfer (ICT) is intense, the two‐photon absorption (TPA) response is also strong.^[^ ^32^, ^33^ ^–^ ^34^ ^]^ High electron‐donating or electron‐withdrawing tendency and *π*‐conjugated structure of substituents that exhibit high charge transfer rates contribute to the TPA performance.^[^ ^35^, ^36^, ^37^ ^–^ ^38^ ^]^ Therefore, investigating the effects of parameters such as the selection of substituents, the type of solvents, and molecular configurations on the photophysical features is a current topic of study for the development of more effective organic materials for their active application areas.^[^ ^39^, ^40^ ^–^ ^41^ ^]^

In addition, density functional theory (DFT) calculations have become an indispensable tool in understanding the effects of substitution on electronic structure and revealing the structure‐property relationship. The attachment of electron donating or electron withdrawing groups to the aza‐BODIPY skeleton at certain positions changes the HOMO–LUMO energy gap, providing redshifted absorption/emission properties. This effect becomes especially pronounced with modifications at the 1,7‐positions.^[^ ^42^, ^43^ ^–^ ^44^ ^]^ The calculations also provide information about the localization of the HOMO and LUMO orbitals for structures such as 1,3,5,7‐tetraphenyl aza‐BODIPY, providing important clues in explaining the light absorption and fluorescence properties.^[^ ^45^ ^]^ In addition, the “push–pull” effects caused by phenyl groups enable aza‐BODIPYs to exhibit effective photophysical responses in the NIR region, making them ideal for therapeutic applications.^[^ ^20^ ^,^ ^46^ ^,^ ^47^ ^]^ In summary, DFT‐based calculations provide a strong theoretical basis for how phenyl substitutions shape the electronic and optical behavior of aza‐BODIPY compounds. This approach, when considered together with experimental data, provides strategic guidance for more efficient and targeted photonic material design.

In this study, aza‐BODIPY compounds functionalized with 4‐methoxyphenyl (MOP) (**BOD1**), 2,4‐dimethoxyphenyl (**BOD2**), and 4‐N, N‐diphenylaminobiphenyl (4‐DPA) (**BOD3**) groups, along with their linear absorption, fluorescence, and TPA properties with respect to charge transfer dynamics, were examined in solution. The charge transfer dynamics and TPA properties of the compounds were revealed by ultrafast pump‐probe spectroscopy and open aperture (OA) Z‐scan experiments. Furthermore, DFT calculations were conducted to elucidate the electronic structure and charge transport mechanisms of these compounds.

## 2 Experimental Processes

### 2.1 Synthesis of Materials

**Scheme 1:**
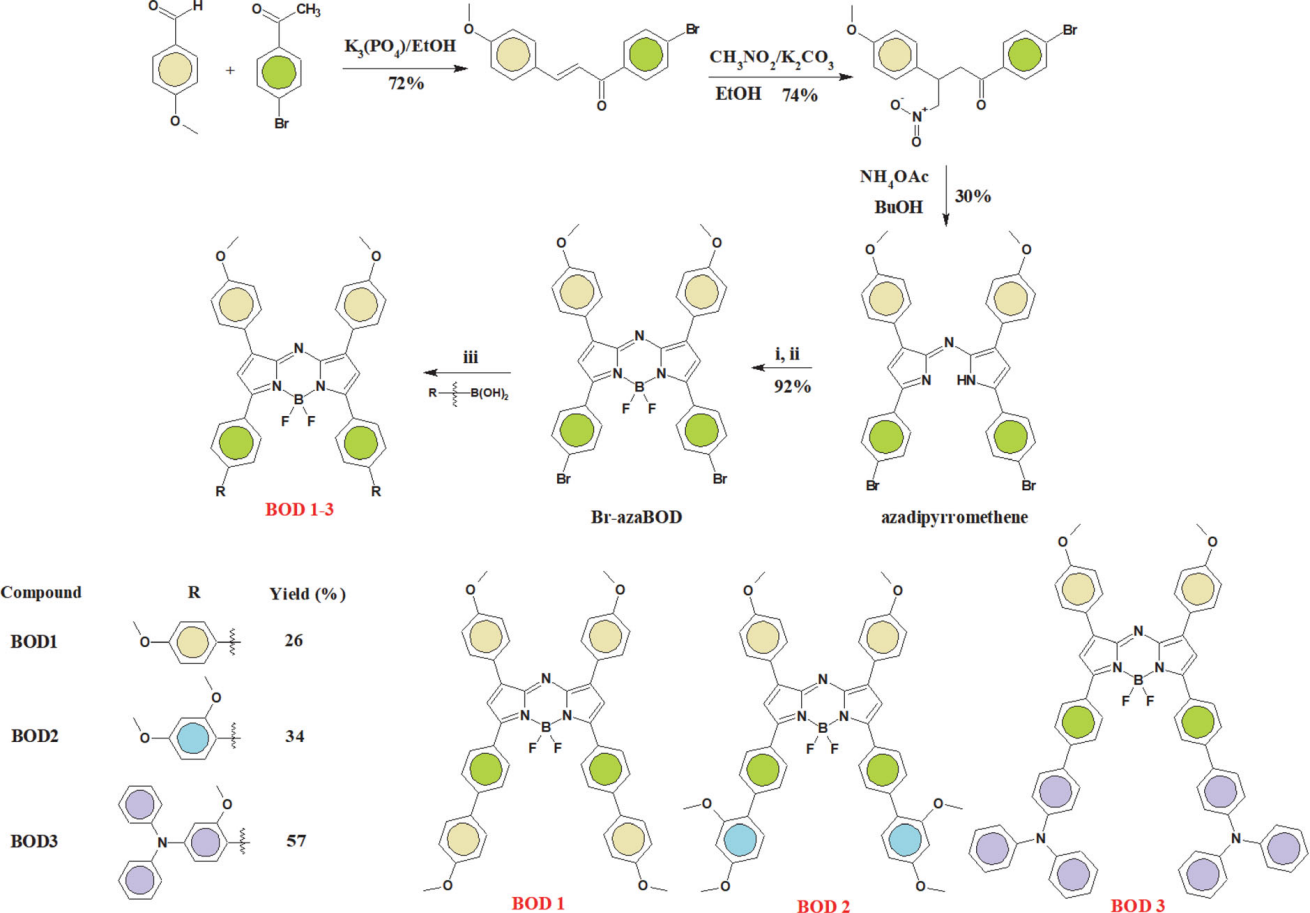
Synthesis and chemical structures of the target Aza‐BODIPYs (**BOD1‐BOD3**). i,ii: BF_3_·OEt_2_, DIEA (Hunig's base), CH_2_Cl_2_, rt., 24 h. iii: Pd(PPh_3_)_4_, K_2_CO_3_, ArB(OH)_2_, H_2_O/EtOH/Toluene (1:1:2, v/v) °C, 24 h.

The synthesis pathway and structure of the resulting aza‐BODIPYs is shown in **Scheme** **1**. The molecules associated with the benchmark set chosen for this investigation have been thoroughly analyzed and documented concerning their synthesis and characterization in our previous study.^[^ ^48^ ^]^ The chalcone intermediate was synthesized in brief via the aldol condensation reaction, employing 4‐methoxy acetophenone and 4‐bromo benzaldehyde as the initial reactants. The chalcone intermediate was subjected to a Michael addition with nitromethane to yield the *γ*‐nitro ketone product, which then underwent treatment with an excess of ammonium acetate to form the aza‐dipyrromethene ligand. Subsequently, the aza‐dipyrromethene was complexed with borondifluoride utilizing boron trifluoride diethyl etherate to obtain the compound Br‐azaBOD. This complex was designed to act as a precursor for the Suzuki‐Miyaura coupling reactions with aryl boronic acids, aimed at extending the conjugation with electron‐donating and *π*‐conjugated substituents. The subsequent stage entailed the employment of aryl boronic acid derivatives in coupling reactions, facilitated by a Pd(0) catalyst, resulting in the synthesis of the target compounds (**BOD1‐BOD3**). The coupling reactions were performed using 4‐methoxyphenylboronic acid, 2,4‐dimethoxyphenylboronic acid, and 4‐(diphenylamino)phenylboronic acid as electron‐donating and *π*‐conjugated substituents.

### 2.2 Optical Measurements

Aza‐BODIPY compounds were prepared in tetrahydrofuran (THF) and chloroform (CHCl_3_) solvents at a concentration of 1.25 × 10^−3^ M. Absorption and fluorescence measurements were made by placing the solutions in 10 mm cuvettes. UV–vIS absorption measurements were made with a scanning spectrophotometer (Shimadzu UV‐1800) and fluorescence measurements were made with a fluorescence spectrometer (Perkin Elmer LS 55). A mode‐locked Ti:Sapphire laser system and an optical parametric amplifier system (Spectra Physics, Spitfire Pro XP, TOPAS) with a 50 femtosecond (fs) pulse duration and 1 kHz repetition rate were used to study charge transfer dynamics with ultrafast pump‐probe spectroscopy and TPA properties with OA Z‐scan technique. A commercial pump‐probe experimental setup from Spectra Physics (Helios) with a white light continuum probe was used to reveal the charge transfer dynamics of the compounds. The pulse duration was measured as 120 fs by cross‐correlation within the pump‐probe setup. Pump‐probe spectroscopy measurements were performed in the time range of 0.1 picoseconds (ps) to 3.2 nanoseconds (ns). Pump wavelengths were determined as the wavelength corresponding to the maximum linear absorbance for each compound. Surface Xplorer (Ultrafast Systems) software was used for analysis of the experimental data. OA Z‐scan experiments were performed with a pulse duration of 50 fs and a pump wavelength of 1000 nm. The laser beam was focused on a solution in a 1 mm‐thick cell by a lens with a focal length of 20 cm. The nonlinear transmittance, T, can be defined in terms of the laser intensity, $I_{0}$ ^[^ ^49^ ^]^

(1) $$T\left(I_{0}\right)=\frac{1}{1+I_{0}\beta l}$$

where β and l are the TPA coefficient and length of optical path, respectively. The β value is obtained from the theoretical fit of the experimental OA Z‐scan data. TPCS, $\sigma_{2}$, is calculated in GM units (1 GM = 10^−50^ cm^4^ s photon^−1^) with the following relation

(2) $$\sigma_{2}=\frac{h\nu \beta }{N_{A}d_{0}\times 10^{-3}}$$

where $N_{A}$ is the Avogadro's number and $d_{0}$ is molar concentration of a solution. According to our previous study, the fluorescence quantum yields (*Φ*_fl_) of the compounds were calculated as 0.31, 0.43, 0.41, and 0.01 for Br‐azaBOD, and **BOD1‐BOD3**, respectively.^[^ ^48^ ^]^

### 2.3 DFT Method

The DFT method is a critical theoretical approach in optimizing the molecular structures and determining the electronic properties of aza‐BODIPY derivatives. In this study, the geometries of the compounds modeled using GaussView 5.0 ^[^ ^50^ ^]^ were optimized in the gas phase using the B3LYP (Becke's Three‐Parameter Hybrid Functional) function and the Def2‐TZVP basis set ^[^ ^51^, ^52^ ^–^ ^53^ ^]^ via Gaussian 09W software.^[^ ^54^, ^55^ ^–^ ^56^ ^]^ The optimizations performed in the gas phase were preferred in order to accurately reflect the intrinsic electronic and geometric structures of the molecules independent of the solvent effects. Following this step, re‐optimization was performed in solvent environments with the same method with the help of the polarizable continuum model (PCM) taking into account the THF and CHCl_3_ solvents used in the experimental studies. Using the optimized structures, the frontier molecular orbitals (FMOs) and NLO properties were calculated in the gas phase, THF, and CHCl_3_ environments; in addition, UV–vis spectra were obtained using the CAM‐B3LYP functional and Def2‐TZVP basis set using the time‐dependent DFT (TD‐DFT) approach to determine the optical properties in THF and CHCl_3_ environments. The CAM‐B3LYP functional gives results that more closely agree with experimental evidence for the optical properties of substituted BODIPY structures, although B3LYP helps to study the electronic structures of multiparticle systems in their ground state.^[^ ^57^, ^58^, ^59^ ^–^ ^60^ ^]^ In addition, in order to evaluate the charge transfer characteristics of the compounds in their excited states, hole–electron analyses were performed from the ground state (S_0_) to the first single excited state (S_1_) using TD‐DFT method. These analyses were based on the wave function data obtained as a result of the TD‐DFT level examination of the optimized structures. Hole–electron distribution analyses were performed using the Multiwfn Software v3.8;^[^ ^61^ ^]^ the obtained results were visualized using the Visual Molecular Dynamics (VMD) software.^[^ ^62^ ^]^ In the maps, green regions ($C_{hole})$ represent hole (positive charge density), and blue regions represent electron (negative charge density) regions ($C_{elec})$. These visuals qualitatively reveal the displacement of the electron within the molecule during excitation and thus enable the evaluation of the ICT character.

## 3 Results and Discussions

### 3.1 Steady‐State Absorption and Fluorescence Properties

**Figure 1:**
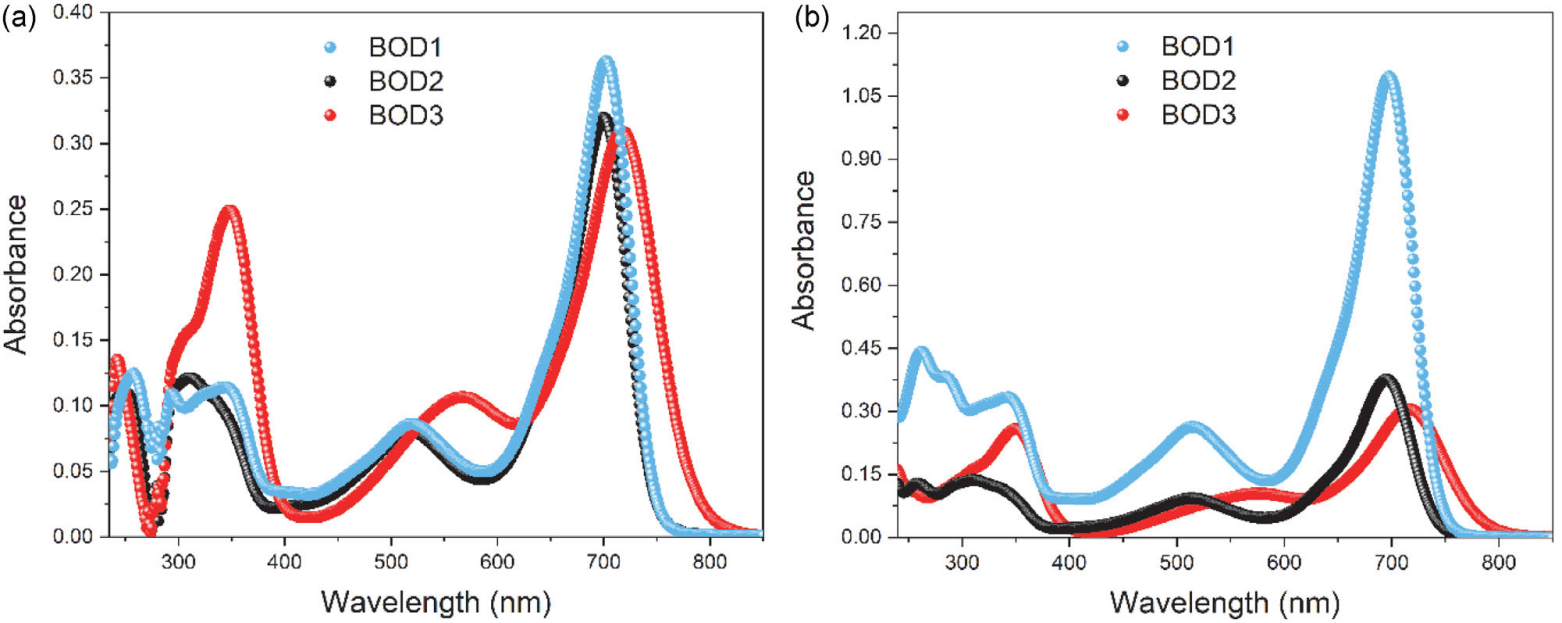
Absorption spectra of **BOD1‐BOD3** compounds in a) THF and b) CHCl_3_ solvents.

The linear absorption measurements of the aza‐BODIPY compounds which were prepared in THF and CHCl_3_ environments were carried out in 200–1100 nm range. **Figure** **1** demonstrates the comparison of the solvent dependent absorption spectra of the compounds. It is seen that the compounds have a broad absorption band from 250 to 750 nm wavelength. The highest absorption peaks were observed at 700 nm for **BOD1** and **BOD2,** and 718 nm for **BOD3** compounds in the THF. These peaks exhibited a hypsochromic shift in the CHCl_3_, occurring at 695 nm for the **BOD1** and **BOD2** compounds, and 715 nm for the **BOD3** compound. Related peaks correspond to the characteristic S_0_ → S_1_ transition of the BODIPY chromophores.^[^ ^63^ ^]^ Further, bathochromic shift in the absorption peaks of **BOD3** compound was observed in both solvents. This can be due to the electron densities delocalized by the BODIPY cores being reduced by the 4‐DPA moiety.^[^ ^64^ ^]^ The observed absorption bands with a weaker intensity in the blue region around 350 nm are attributed to S_0_ → S_2_ transitions for all compounds. **BOD3** compound has the highest absorption peak at 350 nm, while the peaks of **BOD1** and **BOD2** are close.

**Figure 2:**
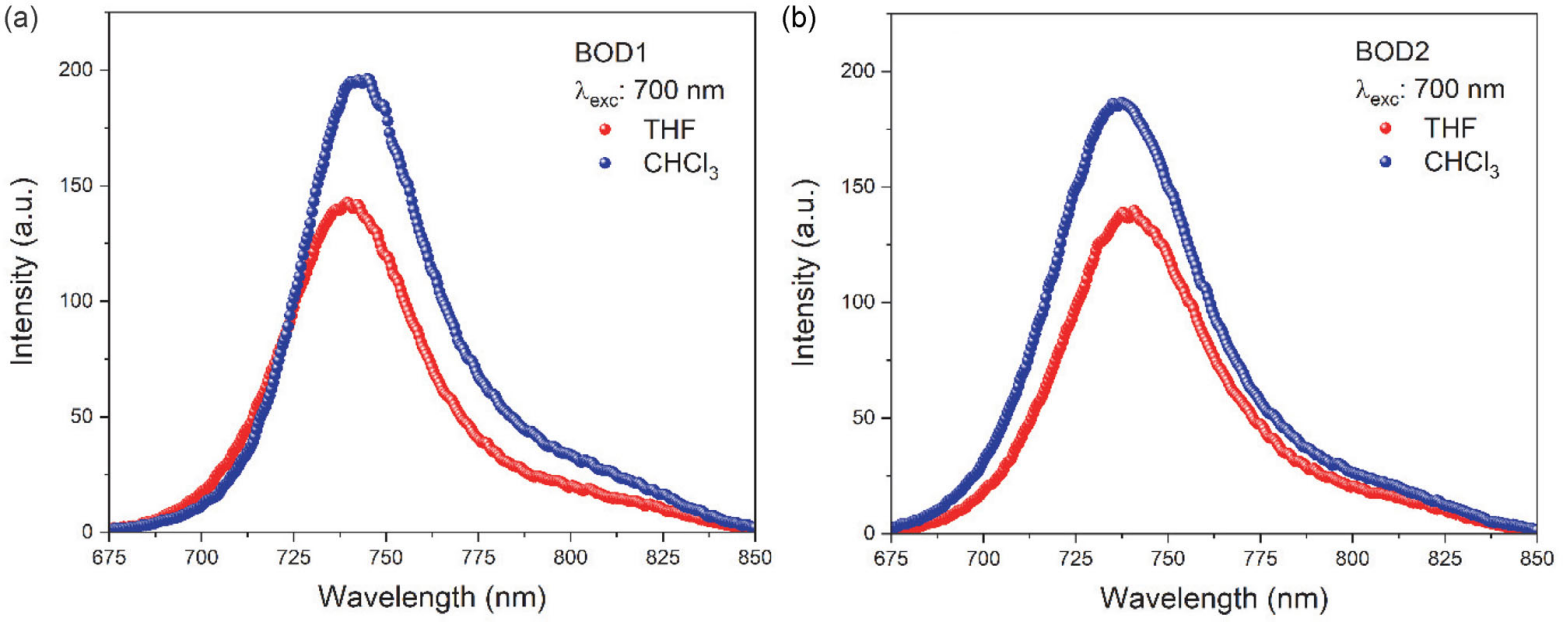
Fluorescence spectra of **BOD1‐BOD3** compounds in a) THF and b) CHCl_3_ solvents.

Fluorescence spectra of the aza‐BODIPY compounds in the THF and CHCl_3_ environments are given in **Figure** **2**. Maximum intensity peaks were observed at 700 nm excitations in both solutions. Peak maximums correspond to 738 and 740 nm in THF solution and 743 and 737 nm in CHCl_3_ solution for **BOD1** and **BOD2** compounds, respectively. Besides, Figure 2a,b indicate the effect of solvent on the fluorescence intensity and wavelengths. **BOD1** and **BOD2** compounds exhibited higher fluorescence intensity in the CHCl_3_ solution and fluorescence wavelengths shifted from 738 to 743 nm and 740–737 nm, respectively. However, the **BOD3** compound did not indicate fluorescence signal. It can be attributed to the quenching of fluorescence by ICT process of strong electron‐donating 4‐DPA moieties. Further, fluorescence intensities of **BOD1** compound are slightly higher than that of the **BOD2** compound in both solutions. In parallel with this situation, it is observed that the excited‐state lifetimes of the **BOD1** and **BOD2** compounds show similar characteristics. The presence of the dimethoxybiphenyl moieties in **BOD2** compound may cause an increment in the charge transfer processes and therefore a little decrement in the fluorescence intensity is observed. To understand the fluorescence quenching mechanisms in detail, charge transfer dynamics in **BOD1**, **BOD2**, and **BOD3** compounds were investigated by ultrafast pump‐probe spectroscopy experiments.

### 3.2 Ultrafast Pump‐Probe Spectroscopy

**Figure 3:**
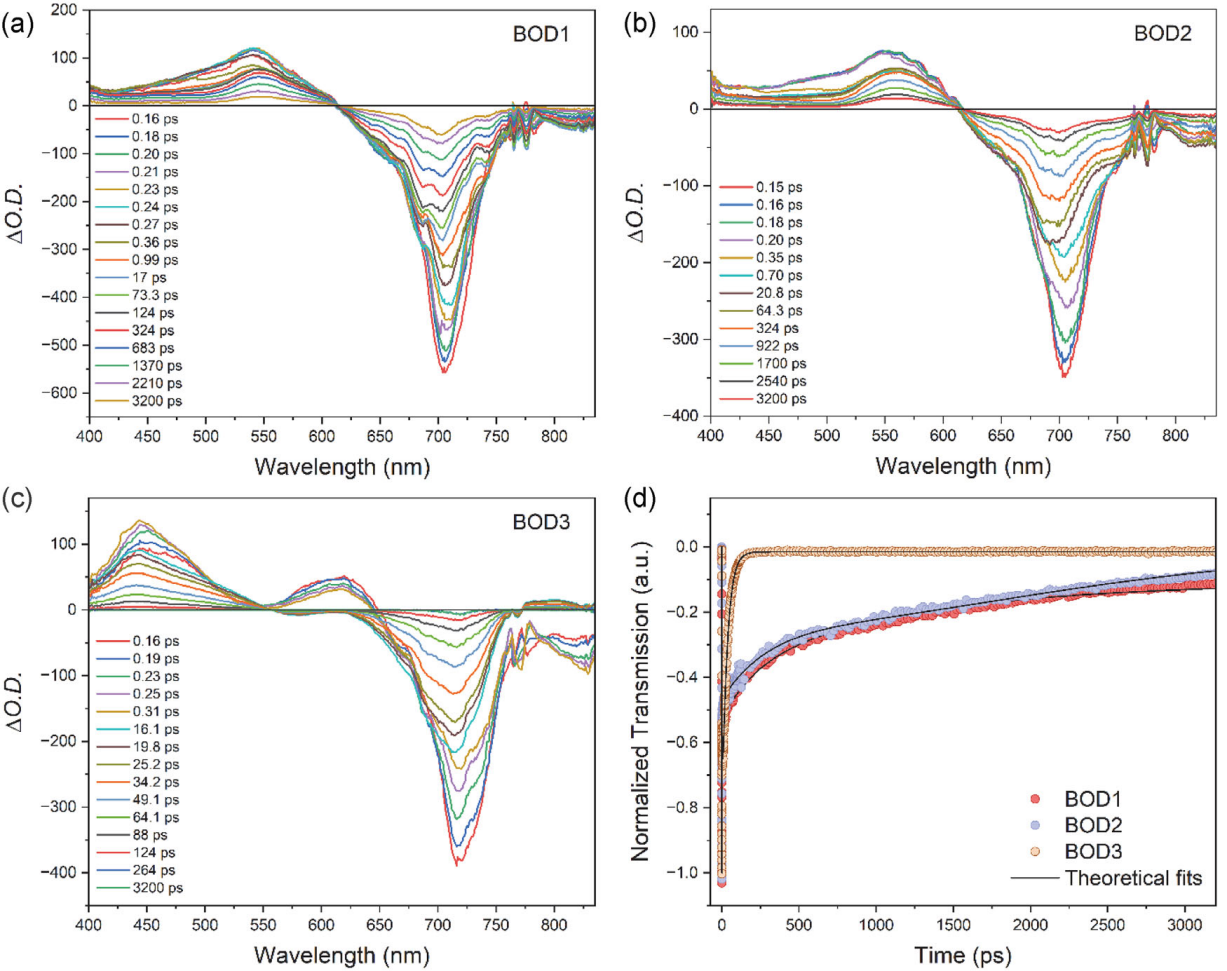
Ultrafast transient absorption spectrums of a) **BOD1**, b) **BOD2**, c) **BOD3,** and d) their decay curves.

Transient absorption spectroscopy measurements in fs time regime were carried out to reveal excited‐state and charge transfer dynamics of the aza‐BODIPY compounds in THF solvent. The pump wavelength was chosen according to the wavelength at which the absorption is maximum (700–720 nm), considering the linear absorption spectra. **Figure** **3** indicates the transient absorption spectra, and the decay kinetics fitted by multiexponential function of the studied compounds. As seen in the figure, there are similar characteristics in the spectral data of the **BOD1** and **BOD2** compounds, but **BOD3** compound shows minor differences. Negative signals around 700 nm in the transient absorption spectra correspond to the depletion of the ground state known as ground‐state bleaching (GSB). The GSB signals decrease from the zero‐time delay until 3 ns in all the transient absorption spectra. Positive signals located in the 400–600 nm range refer to excited state absorption (ESA). In transient absorption spectra of **BOD3**, the ESA signal in the range of 550–650 nm is positive at initial time delay. After a few ps later, it was observed that this ESA signal turns into negative signal corresponding to the saturation of the shoulder of the main absorption band in the linear absorption spectra. Although this signal belongs to the saturation of the LUMO states, it competes with the ESA signal occurring in this wavelength range and eventually dominates the ESA signal. Likewise, the signal above 760 nm is negative initially and turns into positive after a few ps later. At initial time delay, the negative signal is originated from the saturation of the tail of the long wavelength region of **BOD3** compound based on the linear absorption spectra. This signal again competes with the ESA signal and suppressed by the ESA signal.

Figure 3d demonstrates the fitted decay kinetics of the GSB signals in the related wavelengths of the compounds. According to the figure, **BOD1** and **BOD2** have long‐time components as compared to the **BOD3** compound. The reason is that, **BOD3** compound indicates intense ICT driven by the strong electron‐donating nature of its 4‐DPA groups. This is attributed to their extended *π*‐conjugation, which ultimately results in increased ICT and therefore shortened of the excited state lifetime. The excited state lifetimes of the compounds are given in **Table** **1**. The results are also consistent with the intensity of the fluorescence signals, which is proportional to the excited state lifetime.

**Table 1:** Excited state lifetimes of BOD1‐BOD3 compounds.

Compounds	*τ*_1_ [ps]	*τ*_2_ [ps]	*τ*_3_ [ps]
**BOD1**	0.07	496	inf
**BOD2**	0.05	549	inf
**BOD3**	0.06	33	inf

### 3.3 Nonlinear Absorption Properties

The TPA properties of the compounds in THF environment were investigated with OA Z‐scan experiments at 1000 nm wavelength. While the **BOD1** and **BOD3** compounds showed TPA properties, the **BOD2** compound did not show. The reason why **BOD2** compound does not show TPA property can be attributed to the sterical hindrance due to the proximity of the substituents to the BODIPY core.^[^ ^65^ ^]^

**Figure 4:**
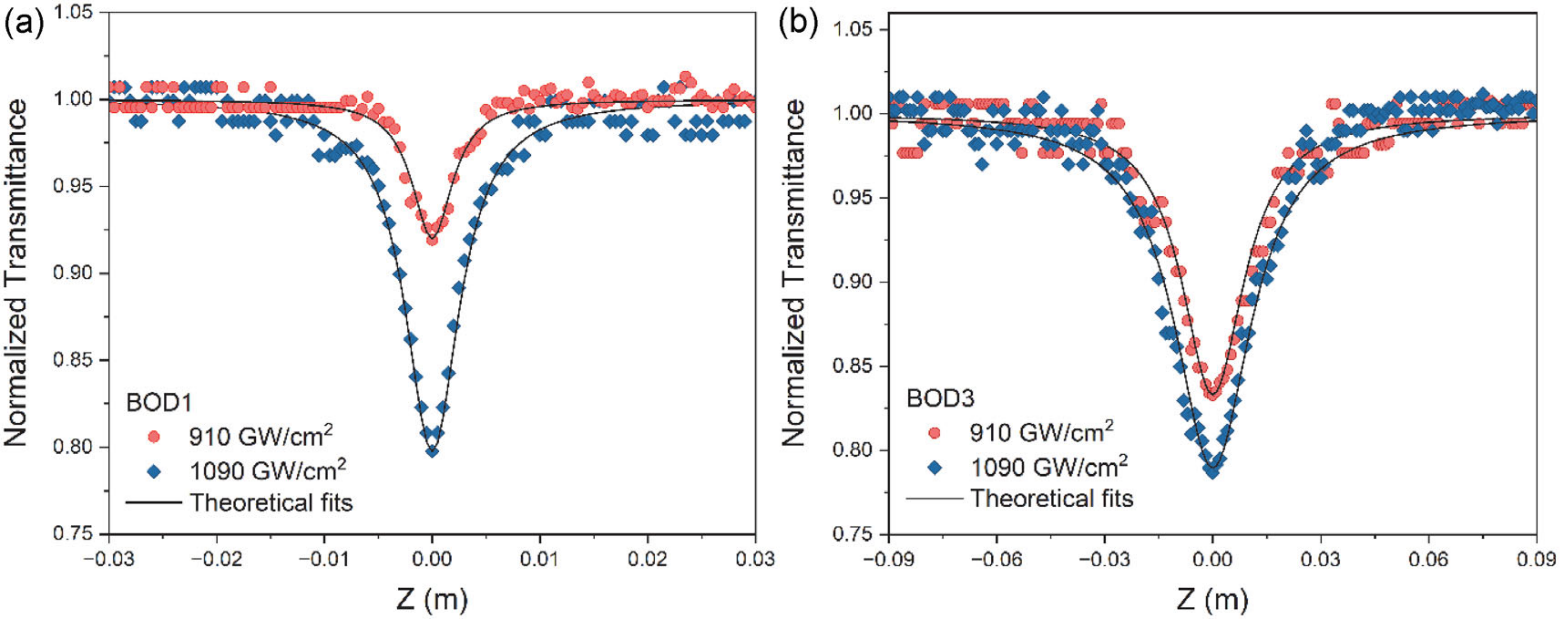
OA Z‐scan results of a) **BOD1** and b) **BOD2** compounds at various intensities.

**Figure** **4** indicates the NIR nonlinear absorption performances of the **BOD1** and **BOD3** compounds at 910 and 1090 GW cm^−2^ intensities. As seen in Figure 4a,b, TPA coefficients increased with increasing intensity in both compounds. At the same intensities, TPA performance of the **BOD3** compound is significantly higher than that of the **BOD1** compound. The TPCS values of the **BOD1** and **BOD3** compounds are listed in **Table** **2**. The highest TPCS was observed to be 269 GM in the **BOD3** compound at 1090 GW cm^−2^ intensity. This is related that the high ICT rate of **BOD3** compound due to the strong electron‐donation of long *π*‐conjugated structured of 4‐DPA moieties. Although the dipole moment also has an effect on TPCS, the ICT change has a greater effect on TPCS, as reported by Xing He et al.^[^ ^66^ ^]^ Moreover, Cesaretti et al. reported the effects of *π*‐conjugation and ICT degree on TPCS and hyperpolarizability properties of organic molecules. It has been demonstrated that in organic molecules, only the change of *π*‐conjugation affects the hyperpolarizability, while the change in both *π*‐conjugation and the degree of ICT strongly affects the TPCS.^[^ ^67^ ^]^ Therefore, higher TPCS values can be observed in the presence of components that increase the *π*‐conjugated structure and ICT ratio of the system, such as 4‐DPA. Besides, the side methoxy donors can cause to decrease TPA performance of **BOD1** compound.^[^ ^27^ ^]^

**Table 2:** Nonlinear optical parameters of the aza‐BODIPY compounds.

Compounds	Concentration [M]	I_0_ [GW cm^−2^]	*β* [m W^−1^]	*σ* [GM]
**BOD1**	1.25 × 10^−3^	910 1090	0.96 × 10^−14^ 2.32 × 10^−14^	2561
**BOD3**	1.25 × 10^−3^	910 1090	9.68 × 10^−14^ 10.2 × 10^−14^	255269

### 3.4 DFT Analysis

#### 3.4.1 Optimization

Optimized structures and related energy values of **BOD1**, **BOD2,** and **BOD3** structures in gas phase, THF, and CHCl_3_ solvents were determined by the method specified in Section 2.3 (Figure S1, Supporting Information). The geometric optimizations of the **BOD1**, **BOD2,** and **BOD3** compounds were performed using the DFT method, and the optimized structures in gas phase are given in Table S1, Supporting Information. This table lists the cartesian coordinates of each atom; these data reflect the 3D positions of the lowest energy structures obtained theoretically. These data provide fundamental information for the analysis of the geometric conformation and electronic properties of compounds. The thermodynamic and electronic properties of the compounds **BOD1**, **BOD2,** and **BOD3** in the gas phase, THF, and CHCl_3_ were also comparatively investigated. This analysis was carried out in order to understand the behavior and stability of the molecules in solvent environments (**Table** **3**). When evaluated in terms of electronic energy, it was observed that solvent environments (especially THF) for all compounds presented lower energy values compared to the gas phase. This observation indicates an enhancement in molecular stability when situated in a solvent environment. For example, while the energy value of **BOD1** in the gas phase was −2542.8093 Hartree, this value decreased to −2542.8287 Hartree in the THF. With an increase in the dielectric constant of the solvent medium, a significant increase in the dipole moment values of the compounds has been observed. For example, while the **BOD1** compound has a dipole moment of 4.19 Debye in the gas phase, this value increases to 5.62 Debye in the CHCl_3_ (*ε *= 4.81) medium and to 5.91 Debye in the THF (*ε *= 7.58) medium. Similarly, an increase in dipole moment values was observed for the **BOD2** and **BOD3** compounds as well, in line with the increase in solvent polarity. These results demonstrate that the molecules are sensitive to the polarity of the solvent medium and that this effect is clearly evident in more polar solvents with increasing dipole moment values. Polarizability values also increased significantly in solvent environments. **BOD3** compound has the highest polarizability due to its large volume diphenylamine groups and this value reaches 1727.76 a.u. in THF environment. This shows that the sensitivity of the molecule to electric fields increases.

**Table 3:** The electronic and thermal properties of the investigated compounds at T = 298.15 K in the gas phase, THF, and chloroform media.

Compounds	Media	Dielectric constant[*ε*]	Electronic energy [Hartree]	Dipole [Debye]	Polarizability [a.u.]	*E*_thermal_ [kcal mol^−1^]	Heat capacity [cal mol.K^−1^]	Entropy [cal mol.K^−1^]
**BOD1**	Gas	–	−2542.8093	4.19	532.924	503.575	192.515	296.069
	THF	7.58	−2542.8287	5.91	694.031	503.413	192.656	296.447
	CHCl_3_	4.81	−2542.8259	5.62	668.872	503.444	192.626	296.410
**BOD2**	Gas	–	−2771.9392	3.71	544.794	547.269	211.148	321.621
	THF	7.58	−2771.9600	4.66	705.757	546.960	211.418	322.293
	CHCl_3_	4.81	−2771.9570	4.49	680.114	546.993	211.399	322.910
**BOD3**	Gas	–	−3348.9350	2.80	1288.302	694.728	257.732	372.502
	THF	7.58	−3348.9556	3.74	1727.756	695.062	259.839	382.111
	CHCl_3_	4.81	−3348.9527	3.60	1659.071	695.099	259.820	381.984

Thermal energy (*E*_thermal_), heat capacity, and entropy values are less affected by the solvent effect; especially the high values of **BOD3** can be associated with the increase in molecular weight and freedom of movement. In terms of entropy, **BOD3** varies between 372.5 (gas) and 382.1 (THF) cal mol K^−^^1^ and shows that the disorder of the system increases with the solvent effect.^[^ ^68^ ^,^ ^69^ ^]^

As a result, THF solvent cause significant changes in the physicochemical properties of BOD derivatives. These results show that appropriate environment selection can be made in optical applications such as TPA and sensor by controlling the behavior of the compounds in solvent environment.

#### 3.4.2 NLO Properties

NLO properties of the aza‐BODIPY derivatives **BOD1**, **BOD2,** and **BOD3** were evaluated via the dipole moment (*μ*), polarizability (*α*), and first hyperpolarizability (*β*) values obtained by theoretical DFT calculations (**Table** **4**) and compared with the experimentally observed TPA properties. According to the experimental OA Z‐scan results, **BOD1** and **BOD3** compounds show TPA properties, while this property is not observed in **BOD2**. This is explained by the steric hindrance caused by the substituents positioned close to the core structure of **BOD2**. The DFT calculation results reveal that the **BOD3** compound exhibits by far the highest *β* value, measured as 20.85 × 10^−28^ and 23.79 × 10^−28^ cm^5^/esu in THF and CHCl_3_ solvents, respectively. This value is consistent with the experimentally observed high TPA coefficient (*β* = 10.2 × 10^−^^14^ m W^−1^) and the highest TPCS (*σ* = 269 GM).

**Table 4:** Total dipole moment (*µ*). polarizability (*α*). and first hyperpolarizability (*β*) of the investigated compounds.

		BOD1		BOD2		BOD3	
	**Solvent**	**THF**	**CHCl3**	**THF**	**CHCl3**	**THF**	**CHCl3**
TotalDipole moment	*μ*_*x*_	0.86	0.80	0.15	0.13	0.92	0.90
	*μ*_*y*_	2.09	2.00	1.72	1.66	1.02	0.97
	*μ*_*z*_	0.54	0.51	−0.62	−0.58	0.51	0.49
	***μ (Debye)***	**5.91**	**5.62**	**4.66**	**4.49**	**3.74**	**3.60**
Polarizability	*α*_xx_	1650.33	1596.98	1642.96	1590.87	2285.01	2206.67
	*α*_xy_	4.47	4.53	−3.64	−4.29	5.35	5.12
	*α*_yy_	1631.82	1555.10	1655.18	1577.01	2128.68	2032.72
	*α*_xz_	1.13	1.49	−20.57	−18.63	8.18	8.41
	*α*_yz_	41.09	38.43	4.94	2.72	106.91	105.00
	*α*_zz_	436.47	413.28	478.13	452.77	799.83	758.73
	***α* (Å** ^**3**^)	**183.50**	**175.93**	**186.34**	**178.66**	**257.26**	**246.63**
First hyperpolarizability	*β*_xxx_	18,611.32	16,692.47	16,348.59	14,719.89	139,363.98	122,173.01
	*β*_xxy_	3919.13	3768.36	−3279.75	−3084.81	29,289.42	24,558.47
	*β*_xyy_	38,733.35	34,721.85	35,130.19	31,431.56	137,068.36	120,208.79
	*β*_xyy_	−4083.86	−3684.16	−5643.45	−5055.36	−18,773.07	−16,491.80
	*β*_xxz_	−462.77	−415.68	1132.71	951.72	−1387.90	−1246.15
	*β*_xyz_	1765.25	1559.52	1818.13	1675.65	−3147.06	−2837.35
	*β*_yyz_	−348.76	−297.62	1.50	−1.84	−210.22	−160.04
	*β*_xz_	−104.58	−92.47	−170.10	−160.08	−1239.48	−1150.18
	*β*_yzz_	175.41	162.47	118.43	116.63	169.99	154.58
	*β*_zzz_	127.27	115.25	28.21	21.04	130.01	121.23
	***β* (cm** ^**5**^ **/esu) × 10** ^**−28**^	**4.43**	**4.95**	**4.03**	**4.50**	**20.85**	**23.79**

The strong electron donating capacity of the long *π*‐conjugation structure of 4‐DPA groups in **BOD3** enhances the ICT of the molecule, which contributes to the superior NLO response observed both experimentally and theoretically. On the other hand, **BOD1** compound exhibited poorer NLO performance with lower *β* and *σ* values, which coincides with the lower hyperpolarizability observed in DFT data. In **BOD2** compound, the low theoretical *β* values and the inability to observe the TPA property experimentally due to structural hindrances reveal a strong agreement between the theoretical and experimental results. As a result, the NLO parameters obtained by DFT were supported by experimental findings and demonstrated the reliability of DFT methods in understanding such structure‐property relationships.

#### 3.4.3 FMOs

FMOs, HOMO, and LUMO, provide important information about the electronic properties and reactivity of *π*‐conjugated systems. The HOMO and LUMO energy levels and energy gaps ($\Delta E=E_{LUMO}-E_{HOMO}$) of the compounds **BOD1**, **BOD2,** and **BOD3** investigated in this study were calculated at 298.15 K in the gas phase and also in THF and CHCl_3_ solvents. The results are summarized in **Table** **5**. The results show that the solvent effects stabilize both HOMO and LUMO orbitals in all compounds. Polar solvents such as THF and CHCl_3_ lower the energy levels due to dielectric stabilization of the molecular orbitals. Among the three derivatives, **BOD3** has the highest HOMO energy (−5.08 eV) in the gas phase.

**Table 5:** Calculated HOMO, LUMO energy levels (in eV), and energy gaps (ΔE) of BOD1–BOD3 in the gas phase, THF, and CHCl3.

Compound	Phase	HOMO [eV]	LUMO [eV]	Δ*E* [eV]
**BOD1**	Gas	−5.20	−3.18	2.02
	THF	−5.41	−3.41	2.00
	CHCl_3_	−5.37	−3.37	2.00
**BOD2**	Gas	−5.18	−3.14	2.04
	THF	−5.41	−3.40	2.01
	CHCl_3_	−5.37	−3.35	2.02
**BOD3**	Gas	−5.08	−3.20	1.88
	THF	−5.21	−3.42	1.79
	CHCl_3_	−5.19	−3.38	1.81

This is consistent with the presence of a diphenylamine group, which is a strong electron‐donating group. These groups narrow the energy gap by increasing electron delocalization on the *π*‐system (Δ*E* = 1.88 eV), making **BOD3** a promising candidate for optoelectronic applications in terms of lower energy light absorption and improved charge transport properties. In contrast, **BOD1** and **BOD2** contain MOP and 2,4‐dimethoxybiphenyl groups, respectively, and show wider energy gaps (≈2.00–2.04 eV). This suggests that they have weaker electron‐donating character than **BOD3**. These results suggest that the effect of functional groups on the electron structure is significant. In general, the trend in the energy gap (**BOD3** < **BOD1** ≈ **BOD2**) confirms how effective structural modifications are in tuning the electronic properties of such BODIPY derivatives. According to Table 1 and 2, depending on the solvent polarity of HOMO and LUMO energy levels, the stabilization observed especially at the LUMO level has narrowed the HOMO–LUMO energy differences and significantly increased the tendency for ICT. In this context, **BOD3** compound is a prominent candidate for optoelectronic applications with its low Δ*E* value and high polarizability. Especially in THF medium, **BOD3** is a candidate to offer the most suitable electronic/optoelectronic performance compared to other compounds with the lowest energy gap and the highest HOMO level.

**Figure 5:**
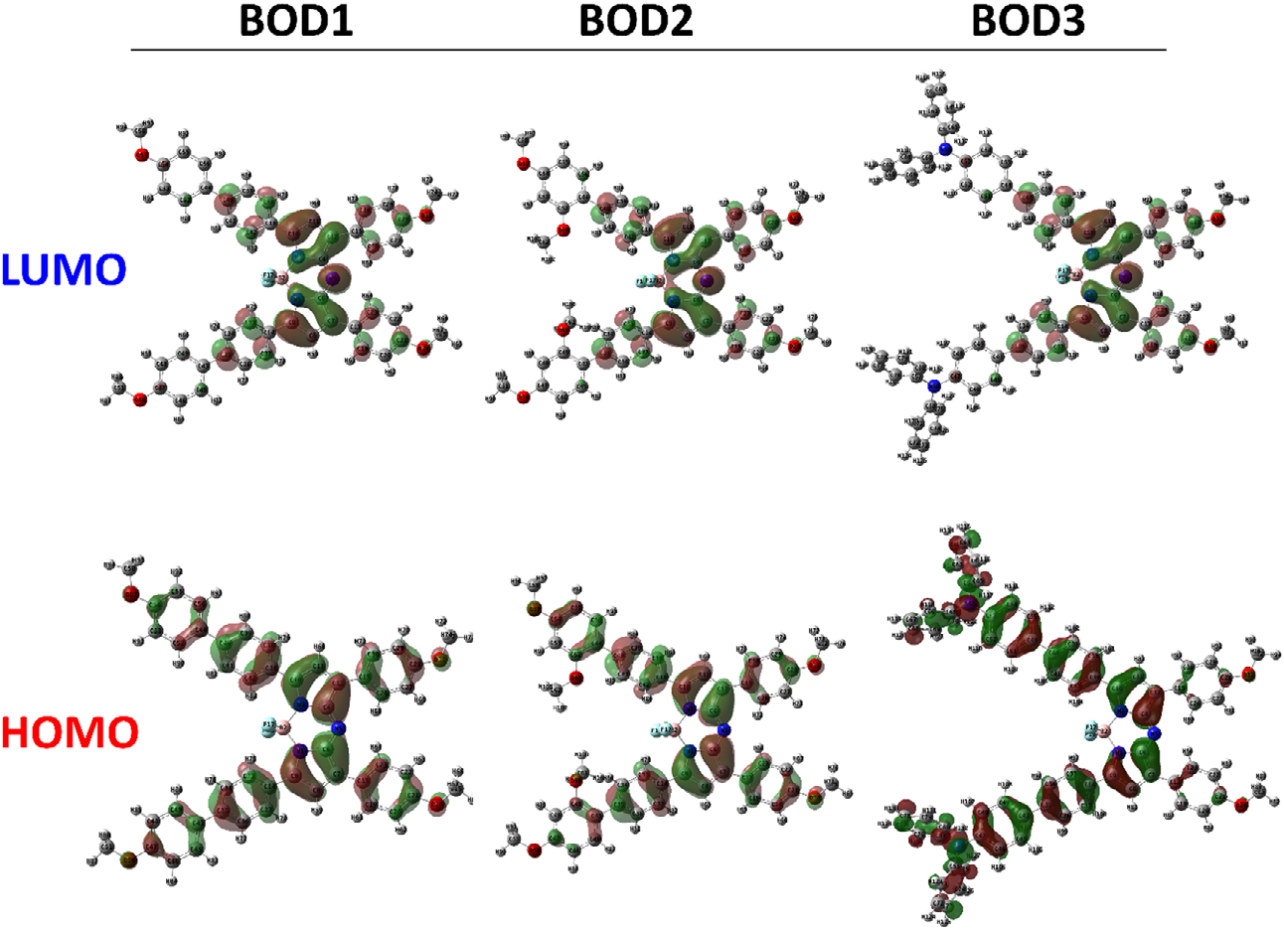
HOMO‐LUMO isosurface distributions of **BOD1**, **BOD2,** and **BOD3** in gas phase.

To obtain a better understanding of the electronic distribution and reactivity of the compounds, the boundary molecular orbitals (HOMO and LUMO) were calculated in different solvent environments. As a result of the analyses, it was observed that the HOMO and LUMO isosurface distributions of the studied compounds were visually similar in gas phase, THF, and CHCl_3_ environments. Although there are slight differences in the respective orbital energy levels, the distributions of the orbitals in space are basically unchanged. Therefore, only the isosurfaces obtained in the gas phase are presented and discussed in this study to avoid repetition, since these structures are representative for all environments (**Figure** **5**).

**BOD1** has symmetrical MOP and 4‐methoxybiphenyl donor groups, which enhances its electron‐rich character. HOMO–LUMO isosurface analysis reveals that the HOMO density is largely concentrated on the methoxybiphenyl group, while the LUMO is localized mainly along the aza‐BODIPY core. This distinct orbital separation indicates that a significant ICT takes place within the molecule. The biphenyl extensions in the donor groups increase the *π*‐conjugation efficiency, which contributes to the moderate to high hyperpolarizability values obtained by calculations. Preservation of the conjugation plane is ensured by linking the donor groups in the 4‐position, thus minimizing steric hindrance. The weak but prominent TPA signal observed experimentally confirms that the ICT mechanism is partially active. In this context, **BOD1** presents a molecular design in which conjugation and electronic dissociation are balanced and exhibits a limited but optimized NLO performance.

**BOD2** contains 2,4‐dimethoxybiphenyl and MOP groups with moderate electron‐donating capacity. HOMO–LUMO isosurface analyses show that both orbitals are localized in similar regions, suggesting that there is no clear donor–acceptor separation and ICT is weak. In particular, the location of the methoxy group at the 2‐position close to the aza‐BODIPY core disrupts the planarity of the molecule and prevents electron delocalization by limiting the *π*‐conjugation system. This structural distortion is consistent with the low hyperpolarizability values obtained by calculations and is confirmed experimentally by the absence of the TPA signal.

**BOD3** contains MOP side groups as well as 4‐DPA group, which is a strong electron donor. According to HOMO–LUMO isosurface analysis, HOMO density is mostly localized on 4‐DPA donor group, while LUMO is localized along the aza‐BODIPY core. This clear donor–acceptor separation indicates the existence of an efficient ICT mechanism. Extended *π*‐conjugation of N,N‐diphenylamino unit with aromatic system provides efficient transfer of electron density to aza‐BODIPY acceptor and largely preserves the planarity of the molecule. This is also consistent with the high hyperpolarizability values and strong TPA signal observed experimentally. Thus, **BOD3** offers a very favorable molecular profile for NLO applications.

#### 3.4.4 UV–vis

The UV–vis spectra of **BOD1**, **BOD2**, and **BOD3** compounds were determined both experimentally and through theoretical calculations, and the data obtained—used to investigate the electronic absorption properties of the compounds—are presented in **Table** **6** using specific abbreviations: $\lambda_{exp}$ (experimental absorbance wavelength), $\lambda_{calc}$ (theoretical absorbance wavelength), *f* (oscillator strength), and $E_{exc}$ (excitation energy). Theoretical calculations were performed by the DFT method, and THF and CHCl_3_ environments were evaluated in order to take into account the solvent effects. In all compounds, the low‐energy fundamental absorption bands observed at long wavelengths correspond to the HOMO → LUMO transition, and these transitions are characterized by high emission strength (*f* ≈ 1.00). This shows that these transitions are allowed and intense electronic transitions. These bands, observed experimentally in the range of 698–719 nm, were theoretically predicted to be between 690–709 nm. A very good agreement was observed between the calculated wavelengths and the experimental values, which supports the validity of the theoretical methods used. The second absorption bands are observed at higher energies (≈300–350 nm) and correspond to transitions involving multiple orbital contributions. For example, in **BOD1** compound, transitions such as HOMO‐1 → LUMO and HOMO‐3 → LUMO contribute by 40–48%. The emission strength values of these transitions (0.4–1.7) are of moderate intensity and can be evaluated as auxiliary absorption bands. **BOD3** compound stands out as the compound with the longest wavelength at 719 nm. This suggests that its HOMO–LUMO gap is narrower and the conjugated system is wider. In addition to the HOMO → LUMO transition, the HOMO‐2 → LUMO transition provides a significant contribution of 21%, indicating that the electronic transitions of this compound are more complex. This indicates that the observed differences in wavelength and energy between THF and CHCl_3_ solvents (<5 nm, <0.02 eV) is minimal within these systems. Moreover, absorption spectra of the compounds demonstrate analogous properties, predominantly unaffected by the surrounding solvent medium. Furthermore, both experimental and theoretical UV–vis analyses revealed that BODIPY derivatives exhibit characteristic *π*–π* transitions and their electronic structures can be reliably predicted by calculations. Especially, the dominance of HOMO–LUMO transitions and high emission strengths indicate that these compounds have potential in optoelectronic applications.

**Table 6:** Theoretical and experimental UV–vis analysis of the investigated compounds.

		Solvent	
Compound	**Property**	**THF**	**CHCl**_**3**_
**BOD1**	$\lambda_{exp}$(nm)	701	698
	$\lambda_{calc}$(nm)	693	695
	*f* (a.u.)	1.03	1.04
	$E_{exc}$(eV)	1.79	1.78
	Major contributions	HOMO → LUMO (96%)	HOMO → LUMO (96%)
	$\lambda_{exp}$(nm)	347	343
	$\lambda_{calc}$(nm)	388	387
	*f* (a.u.)	0.43	0.44
	$E_{exc}$(eV)	3.20	3.20
	Major contributions	HOMO‐3 → LUMO (41%),HOMO‐1 → LUMO (47%)	HOMO‐3 → LUMO (40%),HOMO‐1 → LUMO (48%)
**BOD2**	$\lambda_{exp}$(nm)	699	695
	$\lambda_{calc}$(nm)	690	691
	*f* (a.u.)	1.00	1.00
	$E_{exc}$(eV)	1.80	1.79
	Major Contributions	HOMO → LUMO (96%)	HOMO → LUMO (96%)
	$\lambda_{exp}$(nm)	310	311
	$\lambda_{calc}$(nm)	296	296
	*f* (a.u.)	1.05	1.03
	$E_{exc}$(eV)	4.19	4.19
	Major contributions	HOMO → L + 1 (68%)	HOMO → L + 1 (65%)
**BOD3**	$\lambda_{exp}$(nm)	719	717
	$\lambda_{calc}$(nm)	708	709
	*f* (a.u.)	1.11	1.11
	$E_{exc}$(eV)	1.75	1.75
	Major contributions	HOMO → LUMO (76%), HOMO‐2 → LUMO (21%)	HOMO → LUMO (76%),HOMO‐2 → LUMO (21%)
	$\lambda_{exp}$(nm)	348	350
	$\lambda_{calc}$(nm)	328	327
	*f* (a.u.)	1.76	1.75
	$E_{exc}$(eV)	3.79	3.79
	Major contributions	HOMO‐5 → LUMO (19%),HOMO‐2 → L + 2 (30%),HOMO → L + 1 (40%)	HOMO‐5 → LUMO (21%),HOMO‐2 → L + 2 (14%),HOMO → L + 1 (39%)

#### 3.4.5 Theoretical Investigation of Structural Planarity and ICT Character

**Figure 6:**
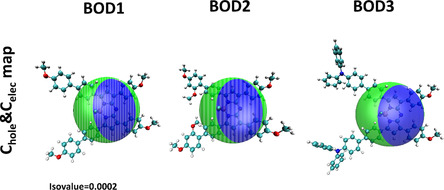
$C_{hole}\&C_{elec}$ distribution maps of **BOD1‐BOD3** compounds in THF solvent.

The structural properties of **BOD1**, **BOD2,** and **BOD3** compounds related to *π*‐conjugation continuity and ICT behaviors in the excited state were theoretically evaluated. For this purpose, dihedral angle values were analyzed on the ground state (S_0_) geometries optimized with DFT (Table S2, Supporting Information). In order for conjugation to be maintained effectively, these torsion angle values are ideally expected to be close to 0^0^ or 180^0^. Deviations from these values indicate that the degree of planarity of the molecules decreases and the *π*‐system continuity is disrupted. According to the obtained results, although there are no major differences between the three compounds, **BOD3** compound stands out with its more symmetric and balanced dihedral angle distribution. The torsion angles, especially in the C11–C12–C15–C30 and C8–C7–C13–C19 regions, being around ±25^0^ indicate that the molecule maintains its general planarity. Similarly, in the **BOD1** compound, the angles ranging from ±25–32^0^ indicate a semi‐planar geometry. However, in the **BOD2** compound, despite similar angle values, it is thought that the planarity is disrupted more due to the distribution of the substituent groups on the molecule and this causes the *π*‐conjugation to be interrupted in some regions. This situation is also consistent with the fact that **BOD2** does not exhibit TPA behavior experimentally. After the structural analyses, the ICT character of the compounds in their excited states was examined via the hole–electron ($C_{hole}\&C_{elec})$ distribution maps calculated with TD‐DFT (**Figure** **6**). In Figure 6, green color represents hole (positive charge density) and blue color represents electron (negative charge density) regions. Hole region shows the area where electron loss is concentrated during excitation, and electron region shows the part that receives electrons. According to hole–electron analysis, **BOD1** compound shows a moderate charge transfer, and hole and electron regions are partially separated. This may contribute to an increase in the polarity of the molecule and an improvement in NLO properties. In **BOD2** compound, hole and electron regions overlap more and ICT character is quite weak. This situation can be associated with the limitation of charge transfer by intramolecular steric hindrances. **BOD3** compound shows the largest spatial separation between hole and electron regions and has a strong ICT character. This structure stands out as the most promising candidate especially for TPA and other NLO applications.

## 4 Conclusion

We attempted to determine the NLO features of aza‐BODIPY derivatives decorated with the 4‐methoxyphenyl, 2,4‐dimethoxyphenyl, and 4‐N,N‐diphenylaminophenyl groups at the 1,7 (distal) positions of aza‐BODIPY core. The effects of the substituents and solvents on the linear absorption and fluorescence properties were investigated. It was found that moieties with strong electron donor properties and long conjugation lengths increase charge transfer in the investigated compounds. Besides, the investigated compounds showed strong TPA properties at NIR wavelength, which is required for two‐photon photodynamic therapy. **BOD3** compound indicated the highest TPCS as 269 GM under the femtosecond laser excitations at 1000 nm wavelength due to the strong charge transfer tendency of 4‐*N, N*‐diphenylaminophenyl moiety. DFT calculations showed that aza‐BODIPY derivatives undergo structural and electronic changes in solvents. Particularly, **BOD3** gained interest due to its narrow HOMO–LUMO energy gap, substantial polarizability, and notable hyperpolarizability. These characteristics were further corroborated by experimental TPA findings. In this context, it was understood that structures with long *π*‐conjugation and electron donating groups are more advantageous in optoelectronic and NLO applications. The high overlap of theoretical and experimental data confirms that DFT approach is an effective tool for predicting the properties of such compounds.

## Conflict of Interest

The authors declare no conflict of interest.

## Supporting information

Supplementary Material

## Data Availability

The data that support the findings of this study are available from the corresponding author upon reasonable request.
