# Supplementary material for: Enhancing Near‐Infrared Two‐Photon Absorption of Aza‐Boron‐Dipyrromethene Compounds Through Intramolecular Charge Transfer Via Electron Donating Substitution
Source: Chempluschem. 2025 Aug 8;90(10):e202500354. doi: 10.1002/cplu.202500354 (PMC12509488; doi:10.1002/cplu.202500354)
Supplement: Supplementary file 1 — Supplementary Material [file CPLU-90-e202500354-s001.pdf]

Supporting Information
For

# Enhancing Near-IR Two-Photon Absorption of Aza-BODIPY Compounds through Intramolecular Charge Transfer via electron donating substitution

Anıl Doğan[a], Halil Yılmaz[b], Ahmet Karatay[a,*], Elif Akhüseyin Yıldız[a], Gökhan Sevinç[c], Huseyin Unver[d], Bahadir Boyacioglu[e,*], Mustafa Hayvali[b,*], Ayhan Elmali[a]

[a]*Department of Engineering Physics, Faculty of Engineering, Ankara University, 06100, Ankara, Turkiye*

[b]*Department of Chemistry, Faculty of Science, Ankara University, 06100, Ankara, Turkiye*

[c]*Department of Chemistry, Science and Literature Faculty, Bilecik Şeyh Edebali University, 11230, Bilecik, Turkiye*

[d]*Department of Physics, Faculty of Science, Ankara University, TR-06100, Ankara, Turkiye*

[e]*Vocational School of Health Services, Ankara University, TR-06290 Kecioren-Ankara, Turkiye*

**Corresponding author:** akaratay@eng.ankara.edu.tr (A. KARATAY), bboyacioglu@ankara.edu.tr (B. BOYACIOĞLU), Mustafa.Hayvali@science.ankara.edu.tr (M. HAYVALI)

**Contents** **Page**

**Scheme S1.** Synthetic pathway of the aza-BODIPYs, **i**: $BF_3OEt_2$, DIEA, $CH_2Cl_2$, rt., 24 h. **ii**: $Pd(PPh_3)_4$, $K_2CO_3$, $ArB(OH)_2$, $H_2O$/EtOH/Toluene (1:1:2, v/v) °C, 24 h……………………………1

**Figure S1.** Optimized structures of **BOD1**, **BOD2**, and **BOD3** in the gas phase, THF, and $CHCl_3$ solvents along with their corresponding optimized energy values…………………………………...2

**Table S1.** Optimized atomic Cartesian coordinates (in Å) of BOD1, BOD2 and BOD3 molecules……4

**Table S2.** Optimized dihedral angles ($^0$) corresponding to substituted positions in aza-BODIPY derivatives in THF solvent……………………………………………………………………………9

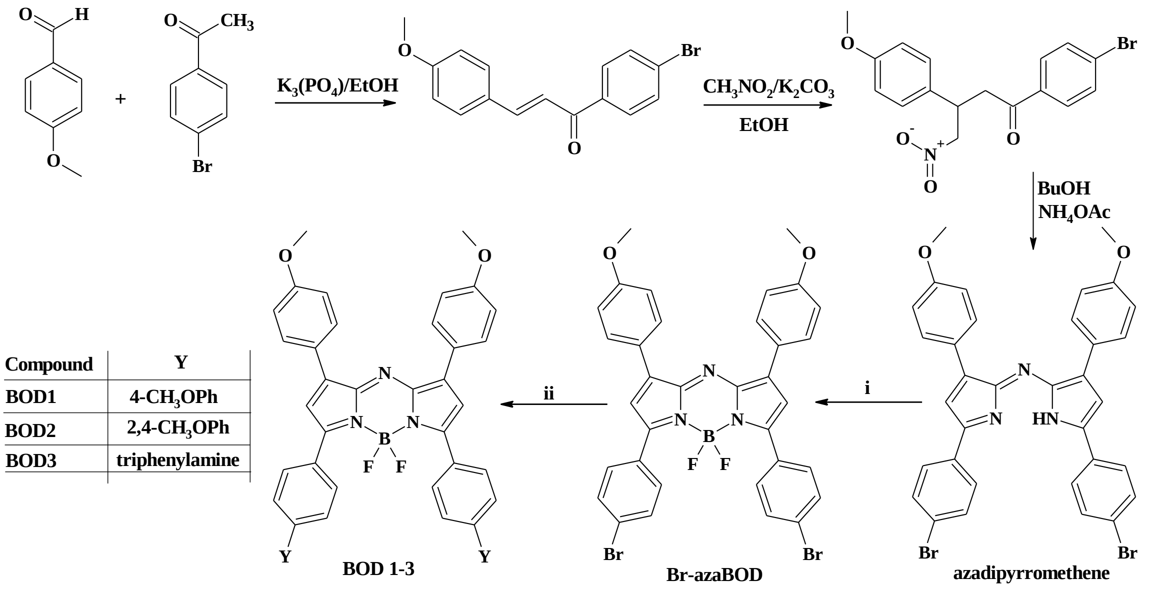


| Compound | Y |
|---|---|
| BOD1 | 4-$CH_3OPh$ |
| BOD2 | 2,4-$CH_3OPh$ |
| BOD3 | triphenylamine |

**Scheme S1.** Synthetic pathway of the aza-BODIPYs, **i**: $BF_3OEt_2$, DIEA, $CH_2Cl_2$, rt., 24 h. **ii**: $Pd(PPh_3)_4$, $K_2CO_3$, $ArB(OH)_2$, $H_2O$/EtOH/Toluene (1:1:2, v/v) °C, 24 h.

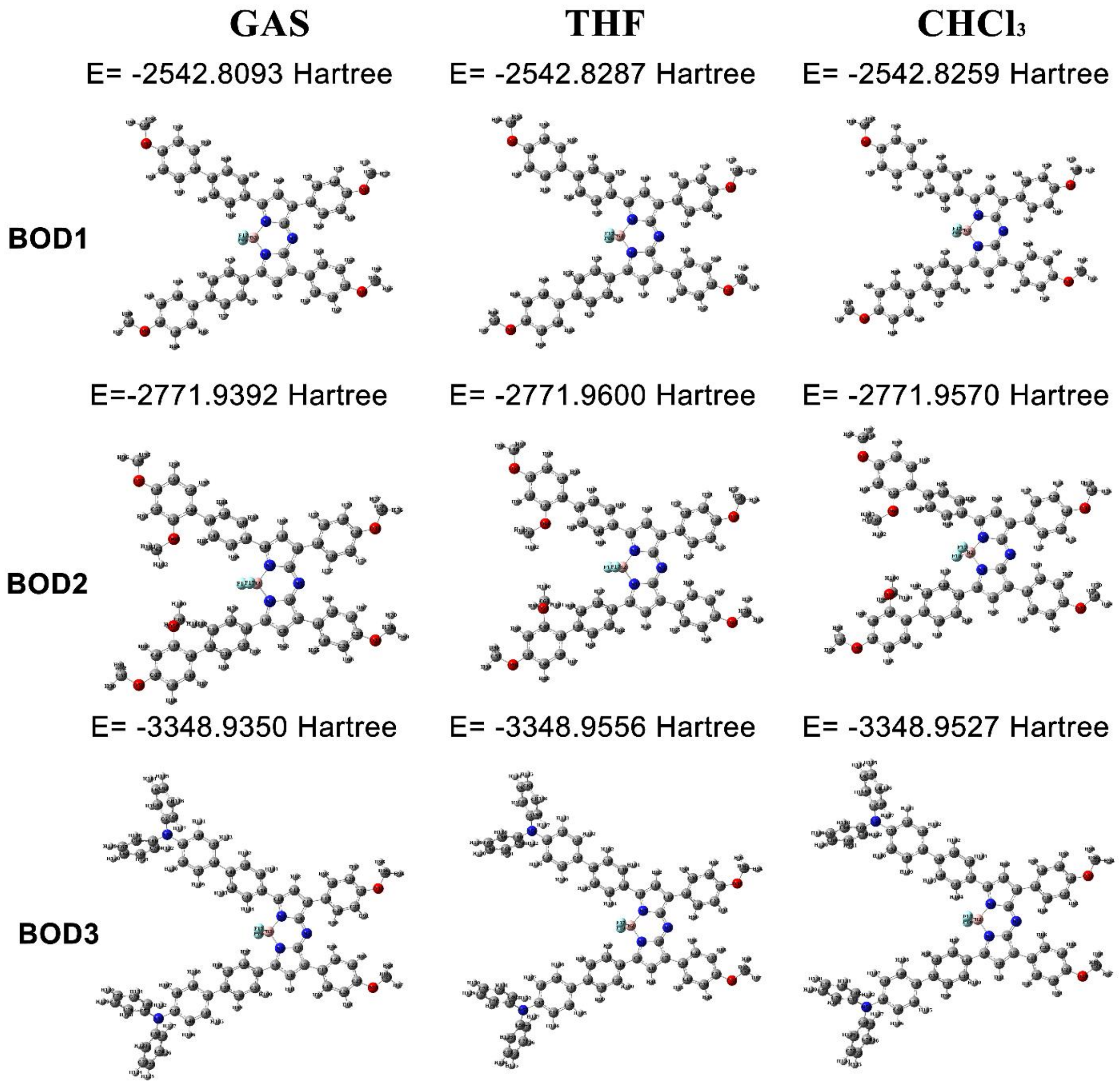


**Figure S1.** Optimized structures of **BOD1**, **BOD2**, and **BOD3** in the gas phase, THF, and $CHCl_3$ solvents along with their corresponding optimized energy values.

**Table S1. Optimized atomic Cartesian coordinates (in Å) of BOD1, BOD2 and BOD3 molecules**

| | BOD1 | | | | BOD2 | | | | BOD3 | | | |
|---|---|---|---|---|---|---|---|---|---|---|---|---|
| **Center Number** | **Atomic Number** | **X (Å)** | **Y (Å)** | **Z (Å)** | **Atomic Number** | **X (Å)** | **Y (Å)** | **Z (Å)** | **Atomic Number** | **X (Å)** | **Y (Å)** | **Z (Å)** |
| **1** | 7 | 1.0532 | -1.3567 | 0.0900 | 7 | 1.5044 | -1.3614 | 0.1785 | 7 | 3.5690 | -1.4461 | 0.1293 |
| **2** | 5 | 0.1852 | -0.0896 | -0.0143 | 5 | 0.6378 | -0.0977 | 0.3016 | 5 | 2.7396 | -0.1576 | -0.0157 |
| **3** | 7 | 1.0942 | 1.1471 | -0.1297 | 7 | 1.5301 | 1.1460 | 0.1482 | 7 | 3.6846 | 1.0473 | -0.1661 |
| **4** | 6 | 2.5084 | 1.0288 | -0.1411 | 6 | 2.9450 | 1.0385 | 0.0872 | 6 | 5.0947 | 0.8880 | -0.1680 |
| **5** | 7 | 3.1461 | -0.1404 | -0.0331 | 7 | 3.5900 | -0.1310 | 0.1240 | 7 | 5.6976 | -0.2964 | -0.0294 |
| **6** | 6 | 2.4712 | -1.2863 | 0.0836 | 6 | 2.9208 | -1.2868 | 0.1690 | 6 | 4.9883 | -1.4184 | 0.1169 |
| **7** | 6 | 3.0116 | -2.6283 | 0.1235 | 6 | 3.4619 | -2.6261 | 0.0844 | 6 | 5.4884 | -2.7743 | 0.1896 |
| **8** | 6 | 1.8955 | -3.4795 | 0.1053 | 6 | 2.3459 | -3.4751 | 0.0002 | 6 | 4.3476 | -3.5921 | 0.1985 |
| **9** | 6 | 0.7019 | -2.7014 | 0.0848 | 6 | 1.1533 | -2.6992 | 0.0685 | 6 | 3.1771 | -2.7799 | 0.1627 |
| **10** | 6 | 0.7887 | 2.5031 | -0.1202 | 6 | 1.2047 | 2.4906 | 0.0325 | 6 | 3.4180 | 2.4116 | -0.1929 |
| **11** | 6 | 2.0072 | 3.2400 | -0.1542 | 6 | 2.4126 | 3.2358 | -0.0944 | 6 | 4.6575 | 3.1120 | -0.2375 |
| **12** | 6 | 3.0933 | 2.3504 | -0.1866 | 6 | 3.5118 | 2.3632 | -0.0580 | 6 | 5.7175 | 2.1908 | -0.2430 |
| **13** | 6 | 4.4210 | -3.0238 | 0.1739 | 6 | 4.8719 | -3.0219 | 0.0856 | 6 | 6.8861 | -3.2102 | 0.2434 |
| **14** | 6 | -0.6466 | -3.2792 | 0.0596 | 6 | -0.1987 | -3.2695 | 0.0405 | 6 | 1.8125 | -3.3174 | 0.1632 |
| **15** | 6 | 4.5157 | 2.6956 | -0.2524 | 6 | 4.9284 | 2.7312 | -0.1479 | 6 | 7.1495 | 2.4938 | -0.3110 |
| **16** | 6 | -0.5399 | 3.1253 | -0.0801 | 6 | -0.1326 | 3.0947 | 0.0302 | 6 | 2.1080 | 3.0713 | -0.1820 |
| **17** | 9 | -0.6700 | 0.0753 | 1.1537 | 9 | 0.0078 | -0.0798 | 1.6307 | 9 | 1.8875 | 0.0703 | 1.1443 |
| **18** | 9 | -0.6897 | -0.2256 | -1.1712 | 9 | -0.4219 | -0.0982 | -0.6762 | 9 | 1.8635 | -0.3046 | -1.1707 |
| **19** | 6 | 4.8007 | -4.3555 | -0.1578 | 6 | 5.2532 | -4.3056 | -0.3979 | 6 | 7.2251 | -4.5591 | -0.0617 |
| **20** | 6 | 6.1323 | -4.7686 | -0.0958 | 6 | 6.5861 | -4.7196 | -0.3873 | 6 | 8.5447 | -5.0095 | 0.0027 |
| **21** | 6 | 7.1369 | -3.8568 | 0.3058 | 6 | 7.5897 | -3.8576 | 0.1140 | 6 | 9.5773 | -4.1193 | 0.3806 |
| **22** | 6 | 6.7874 | -2.5302 | 0.6401 | 6 | 7.2381 | -2.5796 | 0.6009 | 6 | 9.2680 | -2.7765 | 0.6889 |
| **23** | 6 | 5.4448 | -2.1271 | 0.5710 | 6 | 5.8942 | -2.1750 | 0.5822 | 6 | 7.9373 | -2.3359 | 0.6173 |
| **24** | 8 | 8.4317 | -4.3630 | 0.3403 | 8 | 8.8858 | -4.3613 | 0.0870 | 8 | 10.8574 | -4.6620 | 0.4199 |
| **25** | 6 | 9.5285 | -3.4776 | 0.7417 | 6 | 9.9811 | -3.5266 | 0.5884 | 6 | 11.9806 | -3.8009 | 0.8004 |

| | | | | | | | | | | | | |
|---|---|---|---|---|---|---|---|---|---|---|---|---|
| **26** | 6 | 5.5012 | 1.7647 | -0.6858 | 6 | 5.9437 | 1.7801 | -0.4488 | 6 | 8.1102 | 1.5244 | -0.7147 |
| **27** | 6 | 6.8480 | 2.1261 | -0.7681 | 6 | 7.2835 | 2.1621 | -0.5514 | 6 | 9.4671 | 1.8455 | -0.8000 |
| **28** | 6 | 7.2574 | 3.4325 | -0.4151 | 6 | 7.6569 | 3.5109 | -0.3521 | 6 | 9.9117 | 3.1488 | -0.4799 |
| **29** | 6 | 6.2993 | 4.3747 | 0.0215 | 6 | 6.6689 | 4.4745 | -0.0502 | 6 | 8.9787 | 4.1288 | -0.0733 |
| **30** | 6 | 4.9497 | 3.9998 | 0.0962 | 6 | 5.3270 | 4.0785 | 0.0463 | 6 | 7.6188 | 3.7940 | 0.0048 |
| **31** | 8 | 8.6192 | 3.6916 | -0.5318 | 8 | 9.0148 | 3.7880 | -0.4741 | 8 | 11.2809 | 3.3664 | -0.5964 |
| **32** | 6 | 9.1177 | 5.0290 | -0.1997 | 6 | 9.4759 | 5.1676 | -0.2958 | 6 | 11.8151 | 4.6976 | -0.2960 |
| **33** | 6 | -1.7978 | -2.6454 | 0.5946 | 6 | -1.3134 | -2.7029 | 0.7105 | 6 | 0.6826 | -2.6314 | 0.6801 |
| **34** | 6 | -3.0453 | -3.2796 | 0.5649 | 6 | -2.5664 | -3.3273 | 0.6776 | 6 | -0.5827 | -3.2290 | 0.6800 |
| **35** | 6 | -3.2177 | -4.5664 | -0.0058 | 6 | -2.7727 | -4.5399 | -0.0285 | 6 | -0.7973 | -4.5307 | 0.1575 |
| **36** | 6 | -2.0649 | -5.1966 | -0.5429 | 6 | -1.6571 | -5.1022 | -0.7015 | 6 | 0.3344 | -5.2125 | -0.3634 |
| **37** | 6 | -0.8137 | -4.5744 | -0.5057 | 6 | -0.4016 | -4.4874 | -0.6641 | 6 | 1.6031 | -4.6267 | -0.3549 |
| **38** | 6 | -0.6605 | 4.4203 | 0.4971 | 6 | -0.3148 | 4.3306 | -0.6500 | 6 | 2.0169 | 4.3825 | 0.3636 |
| **39** | 6 | -1.8905 | 5.0827 | 0.5485 | 6 | -1.5539 | 4.9779 | -0.6592 | 6 | 0.8068 | 5.0814 | 0.3821 |
| **40** | 6 | -3.0669 | 4.4948 | 0.0145 | 6 | -2.6784 | 4.4335 | 0.0162 | 6 | -0.3798 | 4.5175 | -0.1574 |
| **41** | 6 | -2.9407 | 3.2079 | -0.5676 | 6 | -2.4939 | 3.1993 | 0.6902 | 6 | -0.2823 | 3.2129 | -0.7069 |
| **42** | 6 | -1.7145 | 2.5339 | -0.6120 | 6 | -1.2564 | 2.5443 | 0.6992 | 6 | 0.9230 | 2.5019 | -0.7159 |
| **43** | 6 | -4.5482 | -5.2282 | -0.0414 | 6 | -4.0867 | -5.2398 | -0.0380 | 6 | -2.1446 | -5.1530 | 0.1572 |
| **44** | 6 | -4.3755 | 5.1987 | 0.0623 | 6 | -3.9655 | 5.1797 | 0.0429 | 6 | -1.6643 | 5.2614 | -0.1497 |
| **45** | 6 | -4.6665 | -6.6440 | -0.0432 | 6 | -4.1294 | -6.6603 | -0.0064 | 6 | -2.3103 | -6.5617 | 0.1692 |
| **46** | 6 | -5.9158 | -7.2721 | -0.0749 | 6 | -5.3298 | -7.3758 | 0.0212 | 6 | -3.5792 | -7.1535 | 0.1776 |
| **47** | 6 | -7.0965 | -6.4970 | -0.1085 | 6 | -6.5577 | -6.6784 | 0.0181 | 6 | -4.7503 | -6.3556 | 0.1639 |
| **48** | 6 | -7.0086 | -5.0888 | -0.1091 | 6 | -6.5588 | -5.2715 | -0.0230 | 6 | -4.5976 | -4.9468 | 0.1452 |
| **49** | 6 | -5.7449 | -4.4742 | -0.0748 | 6 | -5.3386 | -4.5716 | -0.0517 | 6 | -3.3242 | -4.3651 | 0.1469 |
| **50** | 8 | -8.2923 | -7.2141 | -0.1391 | 8 | -7.7074 | -7.4645 | 0.0576 | 7 | -6.0458 | -6.9536 | 0.1693 |
| **51** | 6 | -9.5553 | -6.4739 | -0.1644 | 6 | -9.0129 | -6.8011 | 0.0705 | 6 | -2.9113 | 4.5856 | -0.1721 |
| **52** | 6 | -5.5980 | 4.4759 | 0.1025 | 6 | -5.2477 | 4.5460 | 0.0046 | 6 | -4.1258 | 5.2818 | -0.1663 |
| **53** | 6 | -6.8323 | 5.1321 | 0.1477 | 6 | -6.4307 | 5.3012 | 0.0400 | 6 | -4.1483 | 6.6987 | -0.1469 |
| **54** | 6 | -6.8832 | 6.5438 | 0.1519 | 6 | -6.3726 | 6.7078 | 0.1138 | 6 | -2.9088 | 7.3855 | -0.1264 |
| **55** | 6 | -5.6846 | 7.2870 | 0.1120 | 6 | -5.1286 | 7.3661 | 0.1478 | 6 | -1.6999 | 6.6791 | -0.1234 |

| | | | | | | | | | | | | |
|---|---|---|---|---|---|---|---|---|---|---|---|---|
| **56** | 6 | -4.4526 | 6.6112 | 0.0692 | 6 | -3.9573 | 6.5902 | 0.1096 | 7 | -5.3837 | 7.4123 | -0.1508 |
| **57** | 8 | -8.1597 | 7.1040 | 0.1969 | 8 | -7.6098 | 7.3505 | 0.1506 | 6 | -7.1350 | -6.3234 | 0.8659 |
| **58** | 6 | -8.2880 | 8.5624 | 0.2102 | 6 | -7.6386 | 8.8124 | 0.2316 | 6 | -6.2760 | -8.1954 | -0.5189 |
| **59** | 1 | 1.9128 | -4.5565 | 0.1705 | 8 | -5.4258 | -3.1610 | -0.0739 | 6 | -5.5107 | 8.6542 | 0.5632 |
| **60** | 1 | 2.0609 | 4.3159 | -0.2203 | 8 | -5.2596 | 3.1559 | -0.0982 | 6 | -6.5162 | 6.9002 | -0.8752 |
| **61** | 1 | 4.0484 | -5.0678 | -0.4847 | 6 | -5.2548 | -2.5366 | -1.3988 | 6 | -6.1785 | 9.7515 | -0.0288 |
| **62** | 1 | 6.4184 | -5.7831 | -0.3564 | 6 | -6.5405 | 2.4569 | -0.2136 | 6 | -6.3170 | 10.9608 | 0.6751 |
| **63** | 1 | 7.5398 | -1.8159 | 0.9571 | 1 | 2.3635 | -4.5539 | -0.0329 | 6 | -5.7817 | 11.0995 | 1.9719 |
| **64** | 1 | 5.1835 | -1.1085 | 0.8331 | 1 | 2.4517 | 4.3098 | -0.1894 | 6 | -5.1109 | 10.0080 | 2.5607 |
| **65** | 1 | 10.4279 | -4.0928 | 0.6893 | 1 | 4.5009 | -4.9767 | -0.8024 | 6 | -4.9807 | 8.7916 | 1.8676 |
| **66** | 1 | 9.6176 | -2.6279 | 0.0536 | 1 | 6.8738 | -5.6967 | -0.7634 | 6 | -7.8058 | 6.9278 | -0.2950 |
| **67** | 1 | 9.3838 | -3.1155 | 1.7670 | 1 | 7.9897 | -1.9058 | 0.9985 | 6 | -8.9147 | 6.4404 | -1.0094 |
| **68** | 1 | 5.1984 | 0.7619 | -0.9631 | 1 | 5.6306 | -1.1965 | 0.9670 | 6 | -8.7541 | 5.9078 | -2.3048 |
| **69** | 1 | 7.5969 | 1.4176 | -1.1095 | 1 | 10.8818 | -4.1293 | 0.4632 | 6 | -7.4681 | 5.8750 | -2.8817 |
| **70** | 1 | 6.5897 | 5.3806 | 0.3055 | 1 | 10.0675 | -2.6018 | 0.0048 | 6 | -6.3572 | 6.3731 | -2.1782 |
| **71** | 1 | 4.2289 | 4.7315 | 0.4506 | 1 | 9.8370 | -3.2879 | 1.6494 | 6 | -7.0450 | -9.2157 | 0.0877 |
| **72** | 1 | 10.1929 | 4.9876 | -0.3799 | 1 | 5.6672 | 0.7451 | -0.6082 | 6 | -7.2819 | -10.4238 | -0.5920 |
| **73** | 1 | 8.6608 | 5.7889 | -0.8456 | 1 | 8.0545 | 1.4359 | -0.7909 | 6 | -6.7462 | -10.6384 | -1.8782 |
| **74** | 1 | 8.9258 | 5.2679 | 0.8536 | 1 | 6.9305 | 5.5145 | 0.1135 | 6 | -5.9748 | -9.6239 | -2.4813 |
| **75** | 1 | -1.7124 | -1.6725 | 1.0612 | 1 | 4.5849 | 4.8320 | 0.2947 | 6 | -5.7457 | -8.4083 | -1.8130 |
| **76** | 1 | -3.8922 | -2.7742 | 1.0203 | 1 | 10.5558 | 5.1310 | -0.4461 | 6 | -8.4175 | -6.2565 | 0.2733 |
| **77** | 1 | -2.1509 | -6.1674 | -1.0224 | 1 | 9.0187 | 5.8324 | -1.0390 | 6 | -9.4854 | -5.6527 | 0.9607 |
| **78** | 1 | 0.0350 | -5.0809 | -0.9553 | 1 | 9.2528 | 5.5275 | 0.7162 | 6 | -9.2894 | -5.0958 | 2.2409 |
| **79** | 1 | 0.2078 | 4.8943 | 0.9450 | 1 | -1.1954 | -1.7909 | 1.2823 | 6 | -8.0099 | -5.1572 | 2.8300 |
| **80** | 1 | -1.9424 | 6.0514 | 1.0372 | 1 | -3.3970 | -2.8760 | 1.2080 | 6 | -6.9412 | -5.7718 | 2.1540 |
| **81** | 1 | -3.8069 | 2.7337 | -1.0200 | 1 | -1.7783 | -6.0132 | -1.2812 | 1 | 4.3341 | -4.6671 | 0.2922 |
| **82** | 1 | -1.6640 | 1.5624 | -1.0865 | 1 | 0.4199 | -4.9354 | -1.2155 | 1 | 4.7424 | 4.1840 | -0.3299 |
| **83** | 1 | -3.7751 | -7.2631 | 0.0070 | 1 | 0.5081 | 4.7685 | -1.2071 | 1 | 6.4506 | -5.2556 | -0.3699 |
| **84** | 1 | -5.9984 | -8.3549 | -0.0654 | 1 | -1.6584 | 5.9012 | -1.2223 | 1 | 8.7998 | -6.0371 | -0.2380 |
| **85** | 1 | -7.8993 | -4.4703 | -0.1444 | 1 | -3.3285 | 2.7536 | 1.2177 | 1 | 10.0426 | -2.0781 | 0.9875 |

| | | | | | | | | | | | | |
|---|---|---|---|---|---|---|---|---|---|---|---|---|
| **86** | 1 | -5.7000 | -3.3890 | -0.1015 | 1 | -1.1576 | 1.6197 | 1.2533 | 1 | 7.7068 | -1.3049 | 0.8589 |
| **87** | 1 | -10.3360 | -7.2361 | -0.1781 | 1 | -3.1954 | -7.2139 | 0.0306 | 1 | 12.8618 | -4.4427 | 0.7574 |
| **88** | 1 | -9.6622 | -5.8485 | 0.7308 | 1 | -5.3368 | -8.4605 | 0.0600 | 1 | 12.0917 | -2.9677 | 0.0955 |
| **89** | 1 | -9.6280 | -5.8516 | -1.0653 | 1 | -7.4766 | -4.6938 | -0.0337 | 1 | 11.8500 | -3.4148 | 1.8188 |
| **90** | 1 | -5.5855 | 3.3899 | 0.1251 | 1 | -9.7462 | -7.6081 | 0.1088 | 1 | 7.7801 | 0.5234 | -0.9663 |
| **91** | 1 | -7.7624 | 4.5731 | 0.1888 | 1 | -9.1182 | -6.1602 | 0.9548 | 1 | 10.1970 | 1.1072 | -1.1184 |
| **92** | 1 | -5.6963 | 8.3719 | 0.1050 | 1 | -9.1605 | -6.2075 | -0.8406 | 1 | 9.2963 | 5.1334 | 0.1847 |
| **93** | 1 | -3.5429 | 7.2028 | 0.0136 | 1 | -7.4067 | 4.8317 | 0.0066 | 1 | 6.9176 | 4.5552 | 0.3356 |
| **94** | 1 | -9.3607 | 8.7571 | 0.2546 | 1 | -5.0564 | 8.4458 | 0.2125 | 1 | 12.8897 | 4.6215 | -0.4684 |
| **95** | 1 | -7.7955 | 8.9926 | 1.0914 | 1 | -3.0006 | 7.1024 | 0.1630 | 1 | 11.3832 | 5.4529 | -0.9641 |
| **96** | 1 | -7.8682 | 9.0023 | -0.7033 | 1 | -8.6966 | 9.0791 | 0.2469 | 1 | 11.6240 | 4.9691 | 0.7496 |
| **97** | | | | | 1 | -7.1529 | 9.1640 | 1.1504 | 1 | 0.7985 | -1.6452 | 1.1103 |
| **98** | | | | | 1 | -7.1539 | 9.2640 | -0.6430 | 1 | -1.4105 | -2.6818 | 1.1214 |
| **99** | | | | | 1 | -6.0419 | -2.8806 | -2.0823 | 1 | 0.2181 | -6.1965 | -0.8082 |
| **100** | | | | | 1 | -5.3421 | -1.4604 | -1.2356 | 1 | 2.4335 | -5.1740 | -0.7906 |
| **101** | | | | | 1 | -4.2684 | -2.7707 | -1.8159 | 1 | 2.8926 | 4.8412 | 0.8132 |
| **102** | | | | | 1 | -6.2805 | 1.4011 | -0.3053 | 1 | 0.7784 | 6.0619 | 0.8483 |
| **103** | | | | | 1 | -7.1564 | 2.6118 | 0.6810 | 1 | -1.1544 | 2.7530 | -1.1625 |
| **104** | | | | | 1 | -7.0878 | 2.7858 | -1.1059 | 1 | 0.9506 | 1.5176 | -1.1652 |
| **105** | | | | | | | | | 1 | -1.4392 | -7.2096 | 0.2125 |
| **106** | | | | | | | | | 1 | -3.6654 | -8.2351 | 0.2099 |
| **107** | | | | | | | | | 1 | -5.4765 | -4.3104 | 0.1155 |
| **108** | | | | | | | | | 1 | -3.2518 | -3.2820 | 0.1013 |
| **109** | | | | | | | | | 1 | -2.9394 | 3.4996 | -0.1564 |
| **110** | | | | | | | | | 1 | -5.0598 | 4.7286 | -0.1638 |
| **111** | | | | | | | | | 1 | -2.8950 | 8.4709 | -0.1291 |
| **112** | | | | | | | | | 1 | -0.7724 | 7.2449 | -0.1406 |
| **113** | | | | | | | | | 1 | -6.5834 | 9.6561 | -1.0321 |
| **114** | | | | | | | | | 1 | -6.8324 | 11.7959 | 0.2067 |
| **115** | | | | | | | | | 1 | -5.8860 | 12.0367 | 2.5122 |

| | | | | | | |
|---|---|---|---|---|---|---|
| **116** | | | 1 | -4.7005 | 10.0980 | 3.5636 |
| **117** | | | 1 | -4.4761 | 7.9508 | 2.3347 |
| **118** | | | 1 | -7.9356 | 7.3261 | 0.7071 |
| **119** | | | 1 | -9.8999 | 6.4672 | -0.5501 |
| **120** | | | 1 | -9.6122 | 5.5275 | -2.8528 |
| **121** | | | 1 | -7.3304 | 5.4745 | -3.8831 |
| **122** | | | 1 | -5.3723 | 6.3572 | -2.6358 |
| **123** | | | 1 | -7.4511 | -9.0625 | 1.0833 |
| **124** | | | 1 | -7.8745 | -11.1993 | -0.1127 |
| **125** | | | 1 | -6.9266 | -11.5748 | -2.3995 |
| **126** | | | 1 | -5.5624 | -9.7725 | -3.4764 |
| **127** | | | 1 | -5.1635 | -7.6260 | -2.2914 |
| **128** | | | 1 | -8.5739 | -6.6724 | -0.7179 |
| **129** | | | 1 | -10.4654 | -5.6080 | 0.4918 |
| **130** | | | 1 | -10.1152 | -4.6253 | 2.7680 |
| **131** | | | 1 | -7.8456 | -4.7391 | 3.8202 |
| **132** | | | 1 | -5.9625 | -5.8275 | 2.6217 |

**Table description:**

Centre Number: The atomic number of the atom (the index number of the atom within the molecule)

Atomic Number: The atomic number of the atom in the periodic table (e.g. 1 = H, 6 = C, 7 = N, 8 = O, 9 = F, 5 = B)

X (Å), Y (Å), Z (Å): The atom's position in 3D space (in Ångström units)

**Table S2.** Optimized dihedral angles ($^0$) corresponding to substituted positions in aza-BODIPY derivatives in THF solvent

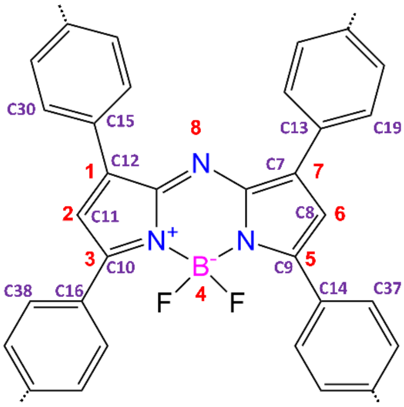


| Compounds | Position | Atomic Numbersı | Optimized Dihedral Angle ($^0$) |
|---|---|---|---|
| **BOD1** | 1 | C11–C12–C15–C30 | 24.46 |
| | 3 | C11–C10–C16–C38 | -32.34 |
| | 5 | C8–C9–C14–C37 | -32.48 |
| | 7 | C8–C7–C13–C19 | 23.52 |
| **BOD2** | 1 | C11–C12–C15–C30 | 24.24 |
| | 3 | C11–C10–C16–C38 | 32.17 |
| | 5 | C8–C9–C14–C37 | -32.68 |
| | 7 | C8–C7–C13–C19 | 24.64 |
| **BOD3** | 1 | C11–C12–C15–C30 | 24.96 |
| | 3 | C11–C10–C16–C38 | -31.49 |
| | 5 | C8–C9–C14–C37 | -32.21 |
| | 7 | C8–C7–C13–C19 | 24.13 |
